# Microbial diversity and bioremediation potential in mangrove sediments—a metagenomic analysis

**DOI:** 10.3389/fmicb.2026.1826301

**Published:** 2026-07-06

**Authors:** Abrar Akbar, Rita Rahmeh, Mohamed Kishk, Batlah Almutairi, Salwa Al-Mutairi, Tahani Al-Waalan, Anisha Shajan

**Affiliations:** Kuwait Institute for Scientific Research, Kuwait City, Kuwait

**Keywords:** bioremediation, mangrove, metagenomics, microbial community, polycyclic aromatic hydrocarbons (PAHs)

## Abstract

Mangroves in Kuwait are exposed to increasing levels of polycyclic aromatic hydrocarbons (PAHs) originating from industrial activities and urban runoff. However, the potential of native mangrove microbial communities for PAH bioremediation has not yet been explored. Sediment samples were collected from three locations (Shuwaikh, Al Khiran, and Sulaibikhat) at varying distances from *Avicennia marina* roots. The concentration of PAHs was determined using GC–MS. To assess microbial diversity, a metagenomic approach was used to evaluate diversity metrics. The statistical analysis included the non-parametric Kruskal-Wallis test (*p* < 0.05) to compare the median values of different groups and the non-parametric PERMANOVA test (*p*-values 0.001–0.009) to assess the differences among groups. The results of the study showed the presence of naphthalene, fluorene, phenanthrene, fluoranthene, pyrene, and chrysene PAHs at varying concentrations across the studied sites. Naphthalene concentrations reached a maximum of 89.64 μg/kg at the Sulaibikhat site and a minimum of 12 μg/kg at the Shuwaikh site. Metagenomic analysis revealed significant differences in microbial diversity between root distances and sites. The rhizosphere samples had higher alpha diversity and richness than the other sediment samples. Beta diversity analysis clustered the samples into groups of sample types and sites. The pairwise comparison between rhizosphere and sediment samples revealed significant differences in microbial communities between rhizosphere and sediment samples in Shuwaikh (*p* = 0.012) and Al Khiran (*p* = 0.007) sites. The heatmap of gene presence/absence revealed the enrichment of genes involved in hydrocarbon degradation (*alkB*, *nahA*, *nahB*, *phnABC*) and plant growth promotion. Functional analysis using KEGG revealed the metabolic capabilities of the isolates, including the presence of peptide/nickel transporters. All bacterial strains were identified by 16S rRNA gene sequencing, and the major groups of bacteria identified were *Pseudomonas*, *Burkholderia*, and *Rhodococcus*, which are known to have the ability to degrade PAHs and promote plant growth. Microorganisms of different species at various sites of Kuwait mangroves showed higher diversity in rhizosphere areas. Microorganisms living in such zones possess the necessary genes to degrade oil as well as for plant growth and thus have the potential for bioremediation of polluted sites by oil. The high level of PAH contamination in the sediment close to the roots of mangroves indicates localized pollution.

## 1 Introduction

Mangrove ecosystems are highly productive ecosystems that support high diversity and act as buffers between the oceanic and terrestrial environments (Das et al., 2024, 2026). Coastal mangrove ecosystems provide important services to humans, including shoreline protection, nutrient recycling, and carbon sequestration (Alshemmari, 2021). Natural filters in mangrove ecosystems remove large amounts human-generated pollutants from the environment (Alshemmari, 2021). Previous studies have identified high levels of persistent organic pollutants in sediments along the coastal areas of Kuwait, including Shuwaikh, Sulaikhat Bay, and Al Khiran, however, the functional performance of these mangrove site is greatly influenced by its salinity, tidal inundation, texture, redox potential and freshwater input (Alongi, 2014; Alshemmari, 2021; Habibi et al., 2025). Among these pollutants, polycyclic aromatic hydrocarbons (PAHs) are a major group of pollutants that have been reported in previous studies (Alshemmari, 2021; Habibi et al., 2025). PAHs are hydrophobic compounds that constitute approximately 20% of the hydrocarbons in crude oil. These compounds are highly persistent in the environment and tend to accumulate in sediments. They pose serious threats to benthic and higher organisms in the food chain (Al-Ghadban and El-Sammak, 2005; Kim et al., 2026).

Mangroves can increase biodiversity, thus the study of the microbial ecology of these ecosystems is highly required (Marcial Gomes et al., 2008). The microbial community within the mangrove ecosystem plays a crucial role in the decomposition of organic matter and nutrient recycling. Consequently, the health of the mangrove system is significantly affected by the microbial community(s) present (Alsharif et al., 2024). These bacteria play key roles in the carbon, nitrogen, phosphorus and sulfur cycling by breaking down organic matters that have previously been deposited into the ecosystem in order to control the release of the essential nutrients in the ecosystem (Birnbaum et al., 2025). High microbial diversity is an indicator of the ability of the ecosystem to adapt and cope with changes in the environment. The mangrove rhizosphere contains an impressive set of copiotrophic bacteria that utilize root exudates for growth and can to degrade refractory organic compounds (Das et al., 2024). For instance, a recent metagenomic study of sediments from the Sundarbans mangrove forest revealed significant differences in taxonomic and functional potential between the microbial communities residing in the rhizosphere of trees and the corresponding bulk sediments. Microbial communities in the rhizosphere of trees harbor an impressive set of genes involved in hydrocarbon degradation and nitrogen fixation (Das et al., 2024).

In addition to identifying genes, metagenomic surveys have been used to study the nutrient cycles of microbial communities in different environments. A metagenomic survey of Rio de Janeiro mangrove sediments by Andreote et al. (2012) reconstructed the nutrient cycles of sedimentary microbial assemblies and recovered several unknown bacterial species, including *Xanthomonadaceae*, *Chromatiaceae,* and *Burkholderiales*. Brazilian metagenomic libraries consist of libraries from very different ecosystems, and recent advances in deep sequencing will allow for a more detailed analysis of the adaptations of sedimentary microbial communities’ (Ben Khedher et al., 2022; Huang et al., 2023; Gao et al., 2024).

Although there is a vast amount of information available on mangrove ecosystems across the world, such as in India, China, and Brazil, there is a knowledge gap regarding the mangrove ecosystems of the Arabian Gulf, particularly Kuwait. The Kuwaiti coastal environment has been exposed to long-term oil pollution resulting from various industrial and shipping activities, as well as historic oil spills. However, the bioremediation potential of mangrove microbial communities native to Kuwait has not been fully explored and characterized (Alshemmari, 2021). Previous studies focused on identifying and quantifying the pollutants in Kuwaiti sediments (Al-Ghadban and El-Sammak, 2005). However, this knowledge gap is even more pronounced given the environmental conditions that prevail in the Arabian Gulf, which are characterized by high salinity levels, extreme temperatures, and very restricted tidal flushing. These conditions may favor the development of specific microbial communities with novel degradation capabilities.

The main objective of this study was to carry out a metagenomic analysis in order to determine the diversity of the microbial populations as well as the sets of functional genes present in sediments collected from three mangrove sites in Kuwait, namely Shuwaikh, Sulaikhat and Al Khiran, and to assess their potential for bioremediation. The results of this study will contribute to a better understanding of mangrove microbial ecology in the Arabian Gulf and provide a scientific basis for the development of bioremediation strategies to reclaim oil-polluted coastal ecosystems in Kuwait.

## 2 Materials and methods

### 2.1 Stages of sampling

#### 2.1.1 Sample collection

Sediment samples were collected from three mangrove sites along the coast of Kuwait: Shuwaikh (29°32′N, 47°84′E), Sulaibikhat (29°33′N, 47°90′E), and Al Khiran (28°67′N, 48°36′E). These locations were selected to represent a gradient of anthropogenic influence, with Shuwaikh and Sulaibikhat situated near urban and industrial areas, and Al Khiran representing a relatively less impacted site. Table 1 presents the grouping and distribution of samples collected from three mangrove-associated locations in Kuwait: Shuwaikh, Al Khiran, and Sulaibikhat. Additionally, rhizosphere samples were collected separately from each study location to evaluate the microbial and environmental characteristics associated with the mangrove root zones. A total of 80 samples were collected across two sampling campaigns conducted in September 2023 and March 2024, representing the dry and wet seasons, respectively. The total samples represent rhizosphere, adjacent (1 m) and bulk (5 m) zones and within each distinct zone per sampling campaign, the raw samples were homogenized and pooled to form the represented replicates analyzed across the nine study groups. Samples were transported to the laboratory on ice and stored at −20 °C for subsequent chemical and molecular analyses.

**Table 1:** Distribution of composite and rhizosphere samples collected from different mangrove-associated locations.

Group	Location	Sampling site description	Sample code
Group 1	Shuwaikh	Adjacent to the mangrove plant	A
Group 2	Shuwaikh	Rhizosphere samples	—
Group 3	Shuwaikh	5 m away from the mangrove plant	C
Group 4	Al Khiran	Adjacent to the mangrove plant	E
Group 5	Al Khiran	Rhizosphere samples	—
Group 6	Al Khiran	5 m away from the mangrove plant	G
Group 7	Sulaibikhat	Adjacent to the mangrove plant	I
Group 8	Sulaibikhat	Rhizosphere samples	—
Group 9	Sulaibikhat	5 m away from the mangrove plant	K

#### 2.1.2 Chemical characterization of polycyclic aromatic hydrocarbon (PAH)

The concentrations of 16 priority PAHs, as designated by the United States Environmental Protection Agency (USEPA), were determined using gas chromatography–mass spectrometry (GC–MS). Briefly, 10 g of freeze-dried sediment was subjected to Soxhlet extraction with a mixture of dichloromethane and hexane (1:1, v/v) for 18 h, following the USEPA Method 3540C. The extract was concentrated using a rotary evaporator and cleaned up using silica gel column chromatography according to the USEPA Method 3630C. The eluate was further concentrated under a gentle stream of nitrogen gas and reconstituted in 1 mL hexane. Analysis was performed on an Agilent 7890B GC system coupled with an Agilent 5977A mass selective detector using an HP-5MS capillary column (30 m × 0.25 mm × 0.25 μm film thickness). The oven temperature program was as follows: initial temperature of 60 °C held for 2 min, ramped to 300 °C at 4 °C per minute, and held for 10 min. Quantification was performed using external calibration standards for each of the 16 PAH compounds, with naphthalene-d8, acenaphthene-d10, phenanthrene-d10, chrysene-d12, and perylene-d12 as internal standards. The detection limits for individual PAHs ranged from 0.1 to 0.5 μg/kg of dry weight.

### 2.2 Bioinformatics analysis

#### 2.2.1 DNA extraction and 16S rRNA gene amplicon sequencing

Microbial DNA was extracted from 0.5 g of each sediment sample using the DNeasy PowerSoil Pro Kit (Qiagen, Hilden, Germany) following the manufacturer’s instructions. The quality and concentration of the extracted DNA were assessed using a NanoDrop ND-1000 spectrophotometer (Thermo Fisher Scientific, Waltham, MA, United States) and a Qubit 4.0 fluorometer with the dsDNA HS Assay Kit (Invitrogen, Carlsbad, CA, United States). We used the primer pair 341F (5′-CCTACGGGNGGCWGCAG-3′) and 805R (5′-GACTACHVGGGTATCTAATCC-3′) to amplify the V3 and V4 regions of the 16S rRNA gene (Klindworth et al., 2012). PCR amplification was carried out in 25 μL reaction mixtures containing 12.5 μL of 2 × KAPA HiFi HotStart ReadyMix (KAPA Biosystems, Wilmington, MA, United States), 0.2 μM of each primer, and 10 ng of template DNA. The thermal cycling program started with an initial denaturation step at 95 °C for 3 min, followed by 25 cycles of denaturation at 95 °C for 30 s, annealing at 55 °C for 30 s, and extension at 72 °C for 30 s. The program ended with a final extension step at 72 °C for 5 min. The PCR products were purified using AMPure XP beads (Beckman Coulter, Brea, CA, United States) and subjected to pyrosequencing using an Illumina MiSeq platform (Illumina, San Diego, CA, United States). Paired-end sequencing runs of 2 × 300 bp were performed on the MiSeq platform to generate reads of approximately 400 bp after quality filtering.

#### 2.2.2 Sequence processing and bioinformatics analysis

Raw sequence data were processed using the Quantitative Insights Into Microbial Ecology 2 (QIIME 2) pipeline (version 2023.5). Demultiplexed sequences were denoised using the DADA2 plugin to correct amplicon errors, remove chimeric sequences, and infer amplicon sequence variants (ASVs). ASVs were taxonomically classified using the SILVA 138.1 reference database with a 99% similarity threshold (Pruesse et al., 2007). Operational taxonomic units (OTUs) were defined at a 97% sequence similarity threshold using a closed-reference OTU-picking approach. Alpha diversity metrics, including ACE, Chao1, Shannon, and Simpson indices, were calculated using the QIIME 2 diversity plugin. Beta diversity was assessed using both weighted and unweighted UniFrac distances, and principal coordinate analysis (PCoA) was performed to visualize community dissimilarities among sample groups (Lozupone and Knight, 2005). The statistical significance of community differences based on location and microhabitat was evaluated using permutational multivariate analysis of variance (PERMANOVA) with 999 permutations.

#### 2.2.3 Functional metagenomic prediction

Phylogenetic investigation of communities by the reconstruction of unobserved states was used to predict metagenomic functional content based on the software package (PICRUSt v1. 0. 0) (Douglas et al., 2020). These predicted metagenomes were then annotated against the KEGG ortholog (KO) database, in which predicted metagenomes were assigned to functional gene categories and biochemical pathways. The focus was placed on genes known to confer the ability to degrade various hydrocarbons (e.g., alkane monooxygenase; *alkB*, naphthalene dioxygenase; *nahA*, *nahB*, etc.). A set of plant growth-promoting genes, including those involved in the fixation of atmospheric nitrogen (*nifH* and *nifK*), solubilization of phosphate (*phoA* and *phoB*), and production of siderophores (*gltB*) to sequester ferric iron, were also investigated.

### 2.3 Microbiological analysis

#### 2.3.1 Isolation and identification of PAH degrading bacteria

Soil samples were homogenized in phosphate-buffered saline (PBS, pH 7.4), and a dilution series (10^−1^ to 10^−6^) was prepared. 100-μL aliquots from each dilution were spread onto nutrient agar (NA) and BHMA (Bushnell-Haas minimal agar (BHMA) supplemented with 1% (v/v) crude oil and 0.025% (w/v) phenanthrene as the sole source of C). Plates were incubated at 30 °C for 2 to 14 days, after which distinctive colonies were picked from the plates and sub-cultured on BHMA several times to obtain pure cultures. Genomic DNA was isolated from pure cultures using a DNeasy UltraClean Microbial DNA Isolation Kit (Qiagen, Hilden, Germany). The 16S rRNA genes from all of the isolates were PCR-amplified using universal primers 27F (5′-AGAGTTTGATCMTGGCTCAG-3′) and 1492R (5′-TACGGYTACCTTGTTACGACTT-3′) and the amplicons were purified and analyzed by Sanger DNA sequencing using an ABI 3730xl DNA Analyzer (Applied Biosystems, Foster City, CA) (Weisburg et al., 1991). All sequences were compared to sequences in the NCBI nucleotide database using the BLAST algorithm, with isolates assigned to a genus if the sequence similarity was 97% or greater.

## 3 Results

### 3.1 Sample collection and characterization

**Figure 1:**
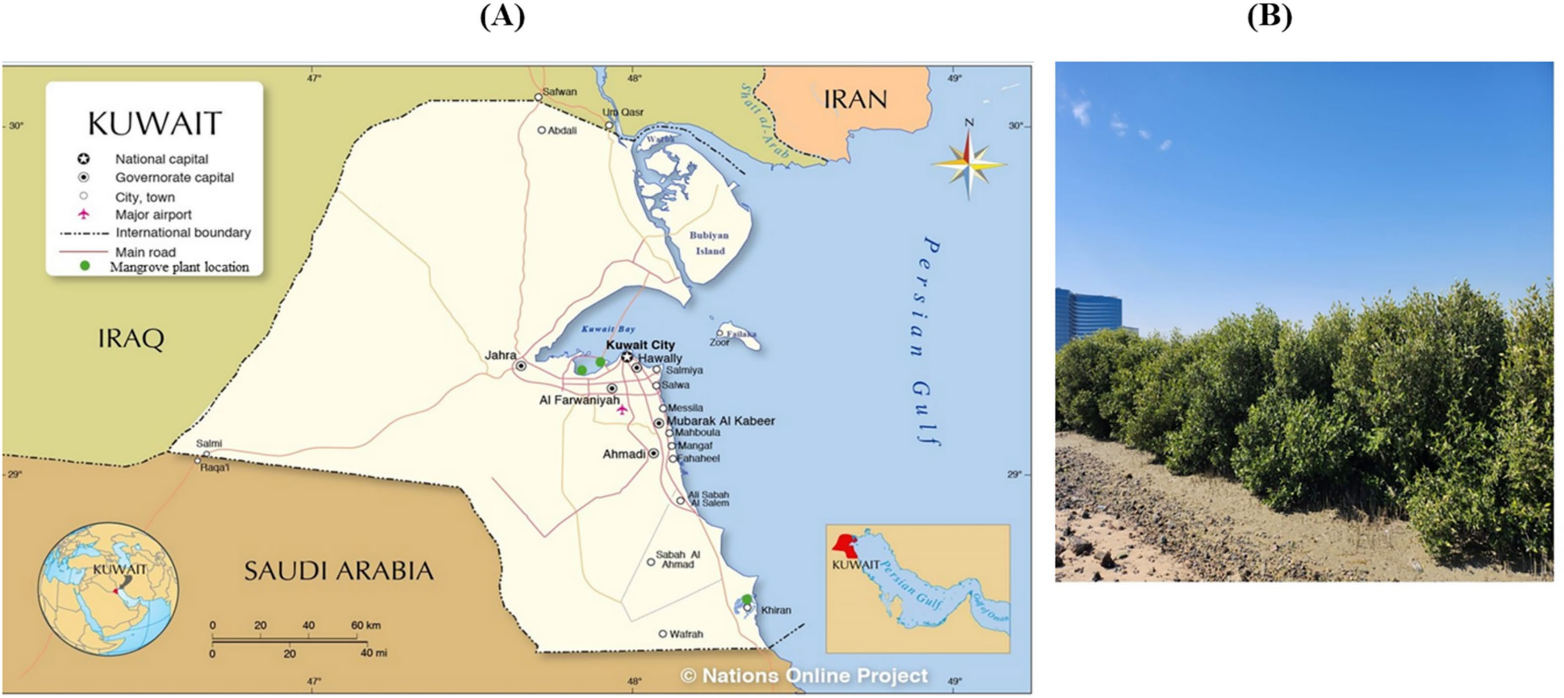
Sampling sites and process. **(A)** Approximate location of sampling site, which includes two points inside the Kuwait Bay and one near the Saudi Arabian border, **(B)** Mangrove plants in Shuwaikh area. Sampling sites are represented as green bullets.

To assess the extent of polycyclic aromatic hydrocarbon (PAH) contamination across the three mangrove sites, the concentrations of 16 priority PAHs were determined in all sediment samples using gas chromatography–mass spectrometry. The analysis revealed that all sediment samples from Shuwaikh, Sulaibikhat, and Al Khiran were contaminated with PAHs, confirming the pervasive nature of these pollutants in Kuwait’s coastal mangrove ecosystems (Figure 1). The most abundant PAH in all samples was naphthalene, with the highest concentration (89.64 μg/kg) in sample I collected from the Sulaibikhat area. Among the tested PAHs, phenanthrene was the second most abundant. The highest concentration of fluorene (5.48 μg/kg) was found in sample C, whereas the lowest concentration (1.68 μg/kg) was found in sample G (Table 2).

**Table 2:** PAH concentration in collected samples.

Target compounds	A	C	E	G	I	K
Naphthalene, 128	37.9 ± 2	12 ± 1.5	22.82 ± 2.65	21.58 ± 2	89.64 ± 5.65	81.27 ± 0.65
Fluorene, 166	4.47 ± 1	5.48 ± 0.7	3.58 ± 0.5	1.68 ± 0.6	4.77 ± 0.4	2.15 ± 0.5
Phenanthrene, 178	7.03 ± 0.65	5.37 ± 1.5	7.63 ± 1	8.08 ± 1.2	15.47 ± 1.6	7.38 ± 1
Fluoranthene, 202	3.2 ± 0.8	0.72 ± 0.5	4.27 ± 0.65	4.28 ± 0.65	6.04 ± 0.6	3.44 ± 0.65
Pyrene, 202	2.5 ± 0.43	0.45 ± 0.65	2.97 ± 0.55	3.01 ± 0.5	4.63 ± 0.63	0.46 ± 0.1
Chrysene, 228	0.77 ± 0.2	ND	ND	0.76 ± 0.52	0.98 ± 0.5	0.30 ± 0.5
Benzo-b-fluoranthene, 252	ND	ND	ND	ND	ND	ND
Benzo-k-Fluoranthene, 252	ND	ND	ND	ND	ND	ND
Benzo-a-Pyrene,252	ND	ND	ND	ND	ND	ND
Indeno-1,2,3-cd-Pyrene, 276	ND	ND	ND	ND	ND	ND
Dibenzo-a,h-Antharacene, 278	ND	ND	ND	ND	ND	ND
Benzo-ghi-Perylene, 276	ND	ND	ND	ND	ND	1 ± 0.2
Benzo-a-Anthracene, 228	ND	ND	ND	ND	ND	ND
Acenaphthylene,152	ND	ND	ND	ND	1.30 ± 0.5	ND
Anthracene, 178	ND	ND	ND	ND	1.7 ± 0.2	ND

All values are in ng/g, ND- not detected. The internal labelling was as follows: (A)- Shuwaikh, adjacent to the mangrove plant, (C)- Shuwaikh, 5 m away from the mangrove plant, (E)- Khiran, adjacent to the mangrove plant, (G)- Khiran, 5 m away from the mangrove plant, (I)- Sulaibikhat, adjacent to the mangrove plant, (K)- Sulaibikhat, 5 m from the mangrove plant. GPS coordinates: (Sulaibikhat: 29.320542, 47.844196; Shuwaikh: 29.33916, 47.9050; and Khiran: 28.647240, 48.3632310).

The concentration of chrysene was detected in samples A, G, and I, reaching a maximum of 0.98 μg/kg in sample I. In addition, benzo-ghi-perylene was detected in sample K at a concentration of 1 μg/kg. The presence of acenaphthylene and anthracene was observed only in sample I, with concentrations of 1.30 μg/kg and 1.7 μg/kg, respectively. In contrast, a group of PAHs, including benzo-b-fluoranthene, benzo-k-fluoranthene, benzo-a-pyrene, indeno-1,2,3-cd-pyrene, dibenzo-a, h-anthracene, and benzo-a-anthracene, were not detected in any of the analyzed samples. This means that they were not present in the studied sediments or that their concentrations were below the detection limit of the method used. Overall, naphthalene was the most abundant contaminant in all samples, followed by phenanthrene. The highest concentration of naphthalene was recorded in the Sulaibikhat area (89.6 ng/g). In addition, for almost all PAH contaminants at all locations, contamination levels were higher in the soil collected adjacent to the mangrove plants than in the surrounding areas.

### 3.2 Isolation and identification of PAH degrading bacteria

**Figure 2:**
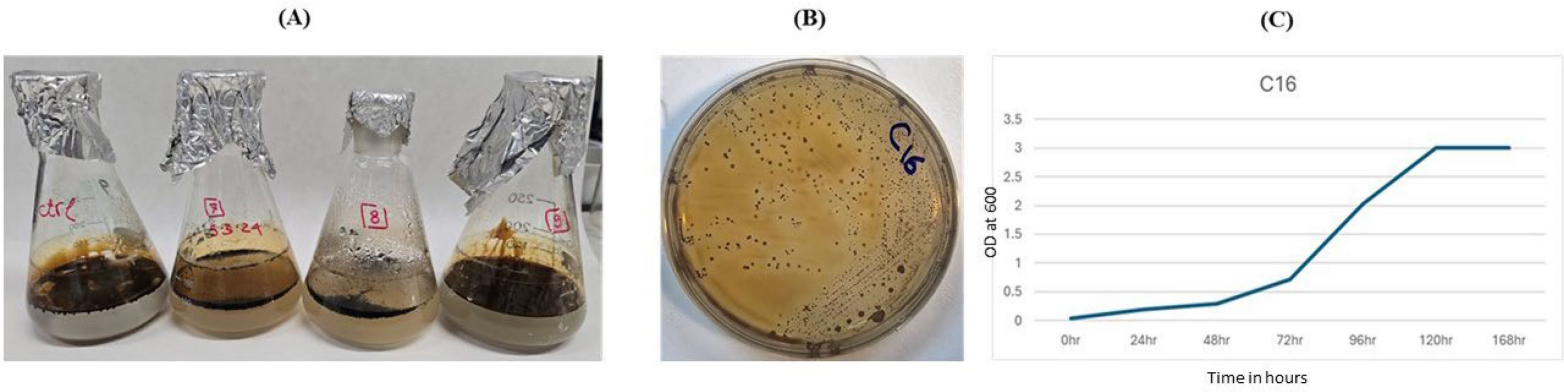
**(A)** Bacterial growth in the presence of crude oil. 1- control (media with crude oil and no sample), 2- bacteria sample 7, 3- bacteria sample 8, and 4- bacteria sample 9. **(B)** *Pseudomonas* sp. (C-16) growth on agar in the presence of crude oil, **(C)** growth curve of *Pseudomonas* sp. (C-16) in the presence of crude oil.

Microorganisms capable of utilizing crude oil and phenanthrene as the sole carbon source were cultured in Bushnell-Haas minimal broth media in the presence of crude oil and phenanthrene at 30 °C for 21 days (Figure 2A). The resulting microbial colonies were isolated for identification and stored at −20 °C for further analysis (Figure 2B). Several bacterial strains were isolated from different soil sediments and labeled C1 to C30. The growth experiment conducted on the isolated bacterial colonies in the presence of crude oil indicated that the isolated microbial strains, namely, C1, C2, C9, C14, C16, and C19, exhibited stable growth rates (Figure 2C).

All 30 isolates were identified using 16S rRNA gene sequencing. The sequences showed 98–100% similarity with known taxa, thus enabling the taxonomical identification of the genus and species (Table 3). *Pseudomonas* was the most abundant genus among the culturable bacteria. In particular, *Pseudomonas stutzeri*, a species capable of degrading a wide variety of hydrocarbons, was identified in several isolates. Other genera isolated were *Burkholderia*, *Stenotrophomonas maltophilia*, *Delftia*, *Bacillus cereus*, *Acinetobacter soli*, and *Rhodococcus ruber*.

**Table 3:** Functional annotation of KEGG Orthologs (Kos) across nine microbial sample groups (groups 1–9).

KO	Description	Group 1	Group 2	Group 3	Group 4	Group 5	Group 6	Group 7	Group 8	Group 9
K02031	ddpD; peptide/nickel transport system ATP-binding protein	0.0040	0.0067	0.0028	0.0030	0.0059	0.0054	0.0077	0.0047	0.0073
K02003	ABC.CD.A; putative ABC transport system ATP-binding protein	0.0048	0.0056	0.0045	0.0044	0.0051	0.0052	0.0058	0.0047	0.0057
K00059	fabG, OAR1; 3-oxoacyl-(acyl-carrier protein) reductase (EC:1.1.1.100)	0.0040	0.0035	0.0026	0.0057	0.0031	0.0059	0.0073	0.0056	0.0038
K02032	ddpF; peptide/nickel transport system ATP-binding protein	0.0031	0.0058	0.0022	0.0026	0.0052	0.0047	0.0065	0.0043	0.0062
K01990	ABC-2.A; ABC-2 type transport system ATP-binding protein	0.0042	0.0025	0.0035	0.0038	0.0022	0.0050	0.0042	0.0046	0.0029
K02483	K02483; two-component system, OmpR family, response regulator	0.0034	0.0031	0.0047	0.0041	0.0034	0.0032	0.0030	0.0036	0.0033
K21023	mucR; diguanylate cyclase (EC:2.7.7.65)	0.0050	0.0037	0.0065	0.0033	0.0035	0.0019	0.0022	0.0021	0.0037
K03406	mcp; methyl-accepting chemotaxis protein	0.0021	0.0057	0.0032	0.0019	0.0064	0.0014	0.0027	0.0023	0.0050
K02049	ABC.SN.A; NitT/TauT family transport system ATP-binding protein	0.0031	0.0040	0.0028	0.0031	0.0029	0.0030	0.0044	0.0029	0.0031
K13590	dgcB; diguanylate cyclase (EC:2.7.7.65)	0.0034	0.0040	0.0039	0.0027	0.0039	0.0012	0.0014	0.0015	0.0036
K02013	ABC.FEV.A; iron complex transport system ATP-binding protein (EC:7.2.2.-)	0.0026	0.0028	0.0022	0.0027	0.0022	0.0038	0.0027	0.0029	0.0026
K06147	ABCB-BAC; ATP-binding cassette, subfamily B, bacterial	0.0026	0.0026	0.0027	0.0027	0.0022	0.0029	0.0029	0.0029	0.0026
K07497	K07497; putative transposase	0.0027	0.0018	0.0022	0.0013	0.0029	0.0038	0.0040	0.0032	0.0018
K02028	ABC.PA.A; polar amino acid transport system ATP-binding protein (EC:7.4.2.1)	0.0021	0.0031	0.0018	0.0019	0.0036	0.0025	0.0029	0.0027	0.0032
K02488	pleD; two-component system, cell cycle response regulator (EC:2.7.7.65)	0.0031	0.0031	0.0039	0.0021	0.0029	0.0013	0.0014	0.0012	0.0026
K03088	rpoE; RNA polymerase sigma-70 factor, ECF subfamily	0.0028	0.0015	0.0024	0.0021	0.0014	0.0035	0.0023	0.0024	0.0016
K07636	phoR; two-component system, OmpR family, phosphate regulon sensor histidine kinase PhoR (EC:2.7.13.3)	0.0021	0.0015	0.0027	0.0033	0.0015	0.0021	0.0018	0.0027	0.0017
K13924	cheBR; two-component system, chemotaxis family, CheB/CheR fusion protein (EC:2.1.1.80 3.1.1.61)	0.0031	0.0011	0.0042	0.0020	0.0010	0.0017	0.0023	0.0018	0.0015
K02010	afuC, fbpC; iron(III) transport system ATP-binding protein (EC:7.2.2.7)	0.0017	0.0023	0.0016	0.0022	0.0021	0.0019	0.0031	0.0017	0.0022
K07678	barA, gacS, varS; two-component system, NarL family, sensor histidine kinase BarA (EC:2.7.13.3)	0.0019	0.0020	0.0032	0.0021	0.0019	0.0016	0.0018	0.0018	0.0022

The table lists the top KEGG orthologs based on mean relative abundance, their functional descriptions, and their distribution across groups. These Kos represent key metabolic functions, including transport systems, regulatory proteins, signal transduction pathways, and hydrocarbon degradation. The observed patterns reflect the functional diversity and ecological specialization of microbial communities in mangrove sediment and rhizosphere environments.

### 3.3 Alpha diversity of microbial communities

To assess the microbial diversity of root samples and sediments at various distances from the mangrove trees at three different locations, alpha diversity metrics as observed by OUT, ACE, Chao1, and Fisher’s alpha were determined for nine sample groups (Groups 1–9). In Shuwaikh (Groups 1, 2, and 3), the highest diversity values were found in the rhizosphere (Group 2), followed by 1-m (Group 1) and 5-m (Group 3) sediment samples. This was manifested by significantly higher species richness estimates (ACE, Chao1, Fisher’s alpha) and Shannon index (*p* < 0.05). In Khiran (Groups 4, 5, and 6), the highest diversity was found in the root samples of Group 5 (rhizosphere), whereas the other non-rhizospheric sediment samples (Groups 4 and 6) revealed lower diversity values, especially in terms of species richness estimates (ACE and Shannon index) (*p* < 0.05). At Sulaibikhat (Groups 7, 8, and 9), the highest diversity was found in the root samples of Group 8 (rhizosphere), followed by the 1-m and 5-m sediment samples (Groups 7 and 9). For the three groups of root samples (Groups 2, 5, and 8), the highest diversity was found in Shuwaikh (Group 2), followed by Khiran (Group 5), and the lowest diversity in Sulaibikhat (Group 8). These differences were manifested by the Shannon and Simpson indices (*p* < 0.05). The sediments located 1 m from the mangrove plants (Groups 1, 4, and 7) showed highest diversity values in Khiran (Group 4), followed by Shuwaikh (Group 1), and lowest in Sulaibikhat (Group 7). In contrast, the sediment samples located farther away from the mangrove roots (Groups 3, 6, and 9) showed the lowest microbial diversity, with the lowest numbers of Observed OTUs and the lowest Shannon and Simpson indices (*p* < 0.05).

### 3.4 Beta diversity among microbial communities across different locations and sample types

**Figure 3:**
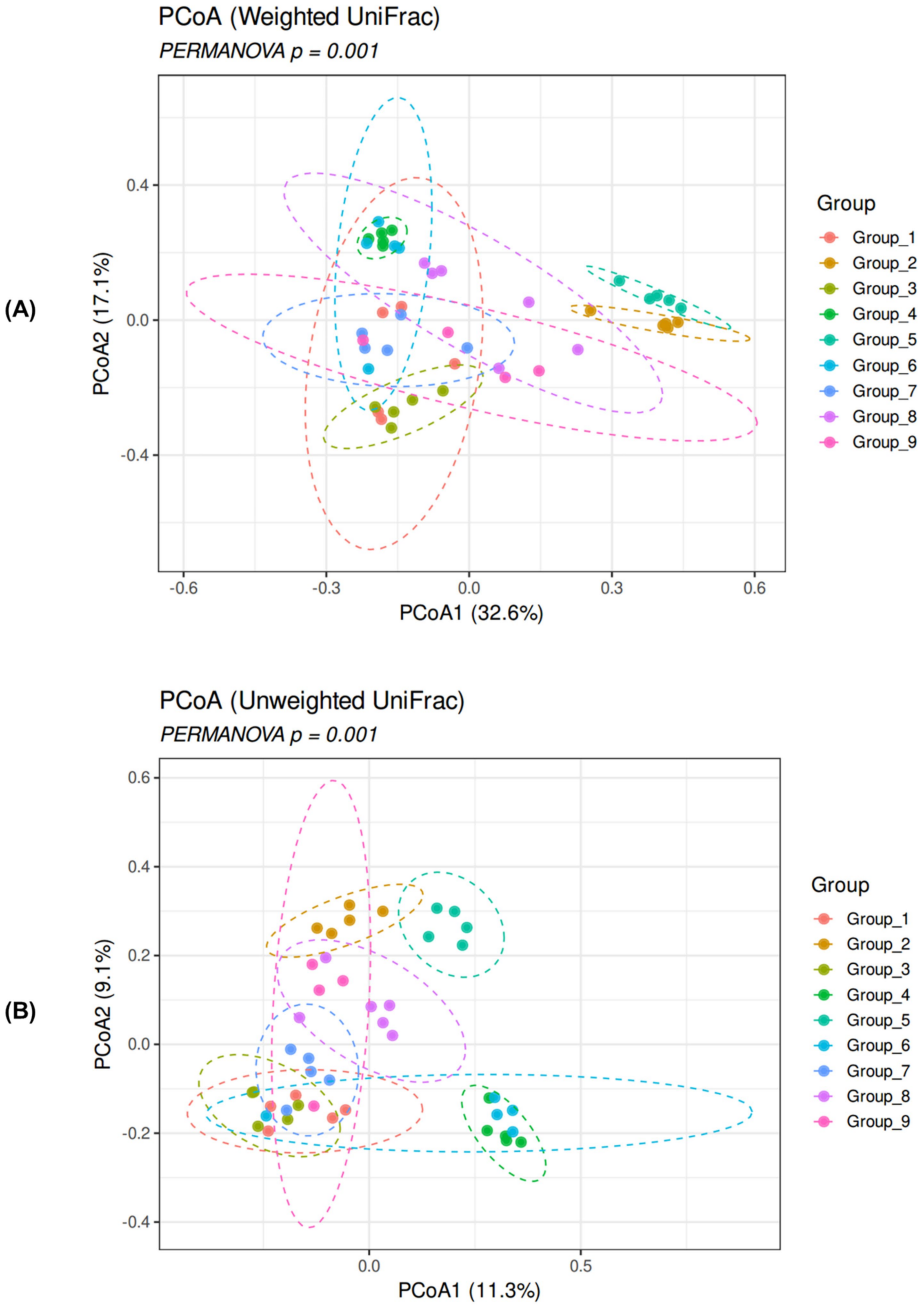
Beta diversity analysis of microbial communities across nine sample groups based on **(A)** Weighted and **(B)** Unweighted UniFrac distances revealed significant community differences across locations and sample types (PERMANOVA *p* = 0.001).

Beta diversity analysis using weighted and unweighted UniFrac distances assessed the differences in microbial communities across the nine sample groups. Principal coordinate analysis (PCoA) visualized sample ordination, showing distinct community clustering influenced by microhabitat and location (Figure 3). Weighted UniFrac PCoA (Figure 3A) indicated that Groups 2, 5, and 8 (rhizosphere samples) clustered together, distinct from sediment samples. Group 5 (Khiran rhizosphere) formed a compact cluster, likely due to root exudate. Sediment samples (Groups 3, 6, and 9) showed high dissimilarity, resulting in an open distribution. Groups 4 and 6 (Khiran sediment) differed from Groups 7 and 8 (Shuwaikh and Sulaibikhat sediment), but no significant difference existed between sediment samples of different locations, whereas rhizosphere samples from the same locations (Groups 2, 5, and 8) showed clear differences. UniFrac distance variation was explained by 32.6% by PCoA1 and 17.1% by PCoA2 (Figure 3A). Unweighted UniFrac PCoA (Figure 3B) showed 11.3% by PCoA1 and 9.1% by PCoA2, with significant group separation (*p* = 0.001), unlike weighted UniFrac PCoA (*p* > 0.05). PERMANOVA (Table 4) showed the highest dissimilarity between Khiran sediment (Groups 3, 6, and 9) and rhizosphere samples (Groups 2, 5, and 8) with R^2^ = 0.776, *p* = 0.007. Significant differences also existed between the Shuwaikh sediment (Group 7) and rhizosphere samples (Group 2), with R^2^ = 0.614, *p* = 0.012. Dissimilarity between sediment samples from different distances showed no significant differences, but multisite comparisons of similar sample types revealed significant differences in microbial communities (*p* = 0.001, R^2^ = 0.410 and 0.446).

**Table 4:** Molecular identification of bacterial strains.

Code	BLAST identification	Score (%)
C-1	*Pseudomonas stutzeri*	100.0
C-2	*Pseudomonas songnenensis*	99.80
C-3	*Burkholderia* sp.	98.00
C-4	*Pseudomonas stutzeri*	100.0
C-5	*Bacillus cereus*	100.0
C-6	*Microbacterium aerolatum*	100.0
C-7	*Kocuria sediminis*	100.0
C-8	*Pseudomonas stutzeri*	99.64
C-9	*Pseudomonas stutzeri strain*	99.86
C-10	*Pseudomonas* sp.	99.64
C-11	*Burkholderia* sp.	99.86
C-12	*Burkholderia cenocepacia strain*	99.86
C-13	*Acinetobacter soli*	99.68
C-14	*Rhodococcus ruber*	100.0
C15	*Micrococcus* sp.	100.0
C-16	*Pseudomonas* sp.	98.70
C-17	*Corynebacterium glyciniphilum*	99.71
C-18	*Stenotrophomonas maltophilia*	99.71
C-19	*Halopseudomonas aestusnigri*	100.0
C-20	*Paracoccus yeei*	100.0
C-21	*Stenotrophomonas maltophilia*	99.93
C-22	*Delftia lacustris*	99.43
C-23	*Delftia tsuruhatensis*	98.00
C-24	*Stenotrophomonas maltophilia*	99.43
C-25	*Delftia lacustris*	99.86
C-26	*Brevundimonas diminuta*	99.14
C-27	*Pseudomonas stutzeri*	99.0
C-28	*Bacillus cereus*	98.4
C-29	*Burkholderia cenocepacia*	99.8
C-30	*Pseudomonas stutzeri*	98.8

### 3.5 Taxonomic profiling of microbial communities

**Figure 4:**
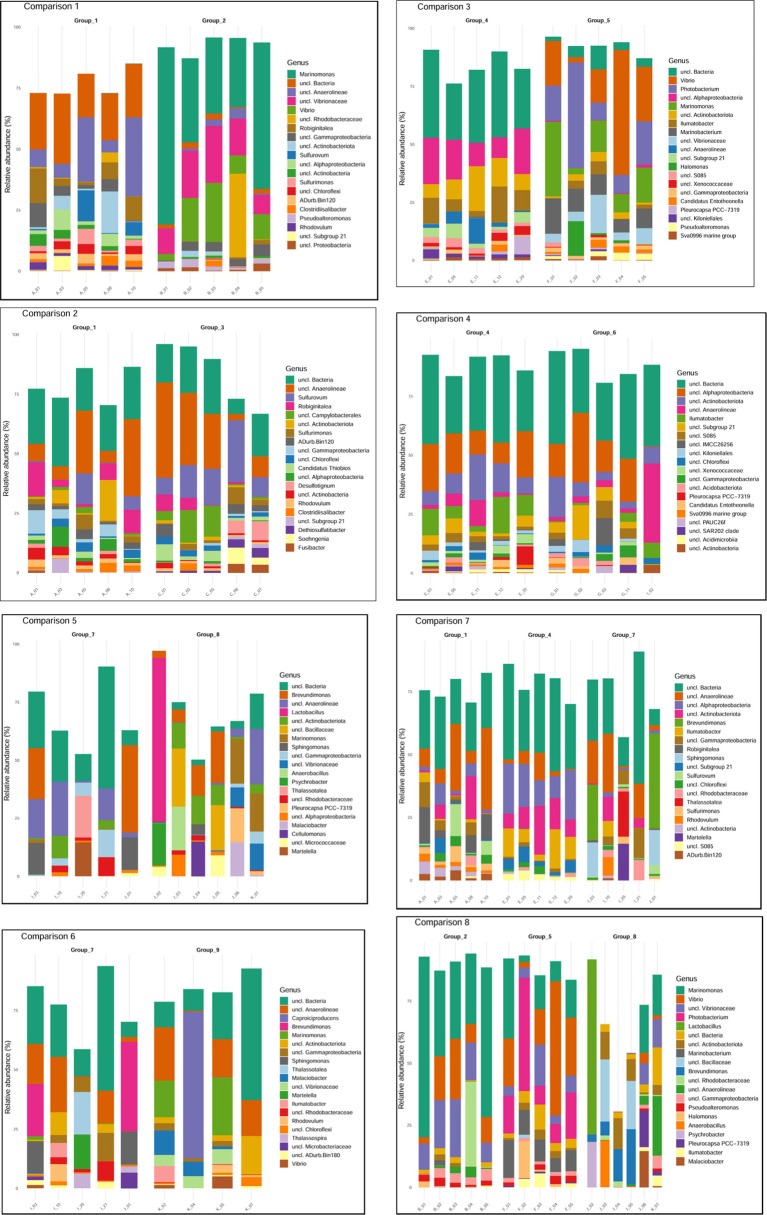
Taxonomic composition of microbial communities at the genus level across the sample groups. Stacked bar charts depict the relative abundance (%) of bacterial genera identified across the different groups (comparisons 1–9). Each bar denotes an individual sample grouped by treatment or condition, with colors corresponding to the different genera, as indicated in the legend. Microbial profiles were analyzed using 16S rRNA gene sequencing, and taxonomic assignment was performed at the genus level of classification. Unclassified taxa are denoted by the prefix “unc.,” followed by the nearest known taxonomic level. Only the 20 most abundant genera across all samples are shown here.

To further analyze community differences, the taxonomic composition of the different sample groups was determined at the genus level. A comparison of the rhizosphere and sediment communities is shown in Figure 4. For Comparison 1 between Group 1 (Shuwaikh rhizosphere) and Group 2 (Shuwaikh sediment), the rhizosphere samples contained highly diverse communities without any single genus constituting more than 50% of the total abundance of the respective samples. The phylum Proteobacteria and Vibrionaceae family were prominently noticed, followed by several other genera, such as *Clostridialesbacter* and *Pseudoalteromonas*. In contrast, the adjacent sediment community (Group 2) was overwhelmingly dominated by *Marinomonas*, which constituted approximately 75% to nearly 100% of the relative abundance in samples B_01 through B_05. We found that root-influenced habitats select for specialized microbes that thrive in these localized environments, whereas adjacent sediments are dominated by other groups of microbes that use available nutrients for growth. In Comparison 1, Group 2 had higher counts of *Marinomonas*, *Vibrio*, unclassified *Vibrioceae*, and *Rhodobacteraceae* than Group 1. In Comparison 2, Group 1 had a diverse suite of unclassified bacteria, including some Robiginitalea, whereas Group 3 had a high number of unclassified *Anaerolineae* and *Clostridiisalibacter.* In Comparison 3, Group 5 had higher counts of anaerobically metabolizing microbes, such as *Vibrio*, *Photobacterium*, *Halomonas*, and *Marinomonas*, than Group 4. In Comparison 4 (Group 4 vs. Group 6), unclassified bacteria were the major component of Group 4, with E_20 having a high amount of *Pleurocapsa* PCC-7319, whereas in Group 6, the L02 sample had high numbers of unclassified *Acidobacteriota*.

In Comparison 5 (Group 7 vs. Group 8), Group 7 showed high microbial heterogeneity. As shown for sample J02, *Lactobacillus* dominated this sample, whereas L21 was dominated by unclassified bacteria, with the other samples of Group 7 showing a mix of mainly *Proteobacteria* and *Actinobacteria*. In Comparison 6 (Group 7 vs. Group 9), the sulfur-oxidizing chemolithoautotroph *Sulfurovum* was abundant in Group 7 for sample L20. In addition, *Thlassospira* dominated sample K04 of Group 9, whereas unclassified *Rhodobacteraceae* were abundant in sample L21 of Group 7. In Comparison 7 (Groups 1, 4, and 7), different microbial profiles were observed in the three groups. Group 1 showed variable abundance of *Rhodovulum* and *Thalassotalea*, with sample J06 being dominated by other microorganisms. For Group 4, unclassified *Gammaproteobacteria* were dominant, whereas *Martelella* was dominant in sample L21 of Group 7. In addition, *Sulfurovum* was clearly dominant in sample L20. In Comparison 8 (Groups 2, 5, and 8), *Marinomonas* clearly dominated the samples of Group 2, as shown for sample J08. In contrast, a high degree of diversity was found for the samples of Group 5, with *Marinomonas, Photobacterium*, *Anaerobacillus*, and *Halomonas* being present in the samples of this group. A clear shift to other microbial communities was observed for Group 8, with samples J_02 and J_03 being dominated by unclassified *Bacillaceae* and *Malaciobacter*, respectively, thereby clearly deviating from the *Marinomonas dominance* found in the other groups. Comparison 9 demonstrated the most pronounced divergence, with Group 9 showing an enrichment of sulfur-metabolizing genera (*Caproiciproducens*, *Marinomonas, Malaciobacter, Robiginitalea*), suggesting biogeochemical sulfur cycling under hydrocarbon stress.

### 3.6 Functional gene profiles for hydrocarbon degradation

**Figure 5:**
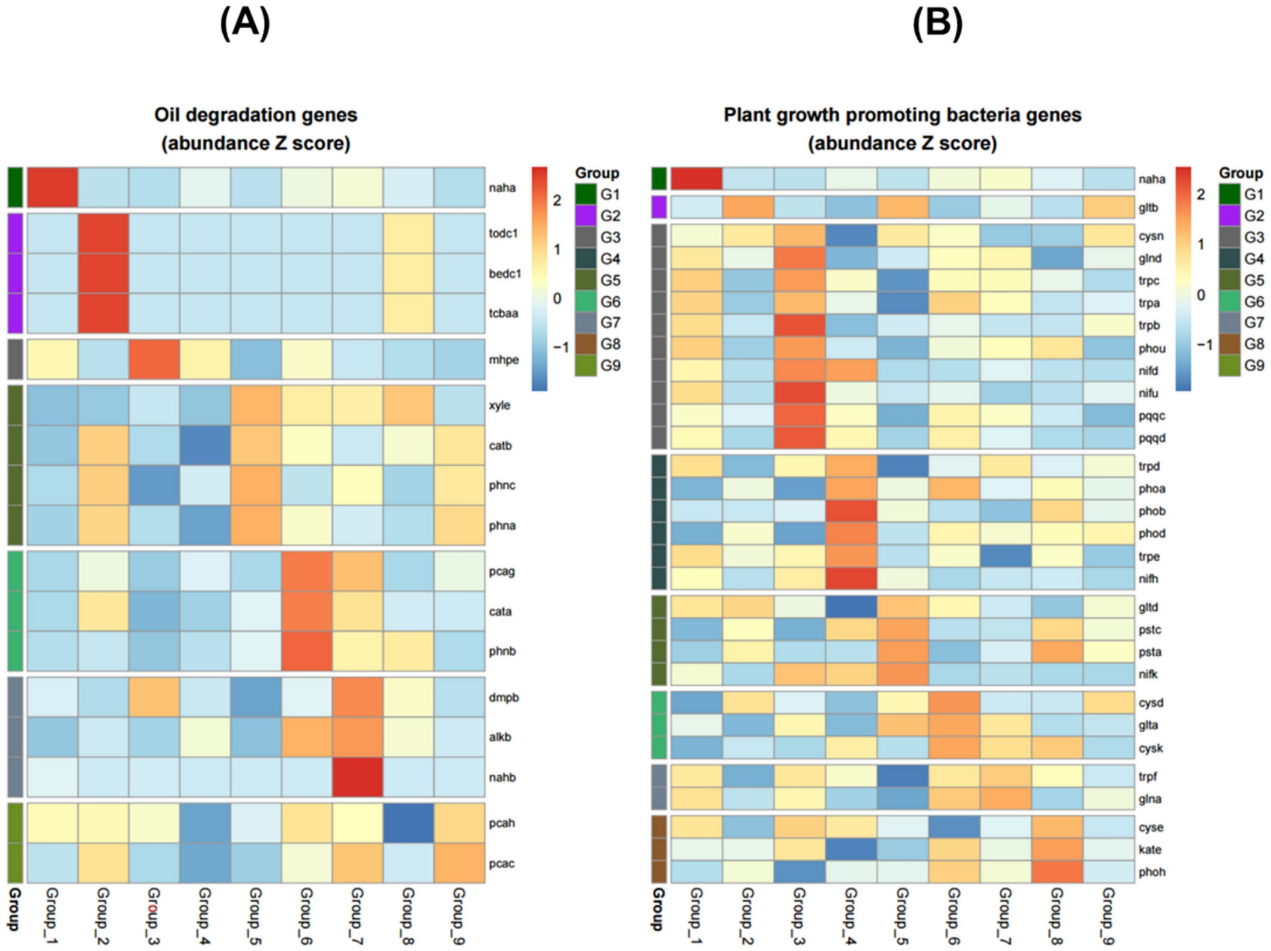
Heatmap representation showing the abundance (Z-score normalized) of functional genes related to oil degradation **(A)** and plant growth-promoting bacteria **(B)** across different microbial groups (groups 1–9). Z-score normalization was applied to each gene across groups to facilitate the comparison of the relative abundances. Functional annotations were derived from KEGG orthology and curated the gene databases. Group labels corresponded to the distinct microbial community profiles obtained from the metagenomic analysis. A heatmap was generated using hierarchical clustering based on Euclidean distance. Each row represents a specific functional gene, and each column corresponds to the microbial group. Warmer colors (red/orange) indicate higher relative gene abundance, whereas cooler colors (blue) indicate lower abundance. The oil degradation genes included those involved in the breakdown of hydrocarbons (e.g., *alkB, nahA, phnA*), whereas the PGPB genes were associated with nitrogen fixation (*nifH, nifK*), phosphate solubilization (*phoA, phoB*), and siderophore production (*gltB, cysK*). Clustering and variation in gene expression patterns illustrate the functional specialization of different microbial communities.

To assess the potential for bioremediation of the studied microbial communities, the abundance of genes involved in the degradation of different types of hydrocarbons was predicted for the nine studied groups of samples using the PICRUSt method. The predicted abundance of genes involved in the initial degradation of alkanes and aromatic hydrocarbons, as well as in subsequent steps of catabolism, when intermediates are transformed into molecules of central metabolism, is presented as a heatmap (Figure 5).

The Z-score-normalized abundance of the predicted oil degradation genes in the nine groups of samples is represented as a heatmap (Figure 5A). Alkane degradation genes, such as *alkB*, which encodes alkane monooxygenase, were highly abundant in Groups 4, 5, and 7, representing the sediments from Sulaibikhat and Al Khiran. This is expected because crude oil consists of aliphatic hydrocarbons, in addition to PAHs, which can be degraded by alkane-degrading bacteria. The abundance of *alkB* genes in the Sulaibikhat sediments was high, especially when compared to other sites. This is consistent with the high PAH levels observed at this site. PAH-degrading genes, including *nahA*, *nahB*, *phnA*, *phnB*, and *phnC*, which are involved in the degradation of different PAHs, were highly represented in the Shuwaikh rhizosphere and adjacent sediments (Groups 1 and 2). The large and small subunits of naphthalene dioxygenase (*nahA* and *nahB*), which are involved in the first step of naphthalene degradation by adding two hydroxyl groups, were the most abundant genes. This is expected because naphthalene is the most abundant PAH at this site. Phenanthrene-degrading genes (*phnA*, *phnB*, and *phnC*), which encode phenanthrene dioxygenases that catalyze the first step of phenanthrene degradation, were also highly represented in the Shuwaikh rhizosphere and adjacent sediments.

Core genes of the aromatic degradation pathway, such as *xylE* (catechol 2,3-dioxygenase), *catA* and *catB* (catechol 1,2-dioxygenase), and *pcaG*, *pcaH*, and *pcaC* (protocatechuate 3,4-dioxygenase, subunits A, B, and C) were detected in most of the groups investigated, especially in Groups 1, 2, 4, and 7. These genes encode enzymes that channel catechol and protocatechuate intermediates into the tricarboxylic acid (TCA) cycle via the meta- or ortho-cleavage pathway. Thus, the investigated microbial communities can complete the mineralization of aromatic hydrocarbons to carbon dioxide and water instead of forming persistent intermediate metabolites. This is a very important aspect for potential bioremediation applications, as it guarantees the detoxification of polluted environments by removing toxic intermediate metabolites.

Figure 5B illustrates the abundance of genes associated with plant growth promotion, including nitrogen fixation, phosphate solubilization, siderophore production, and hormone modulation. Group 3 exhibited upregulation of *gltB, trpC, trpA, trpB, phoU*, and *pqqC/D*, which are linked to tryptophan metabolism, phosphate uptake, and PQQ-mediated redox functions, indicating an active plant growth-promoting community. Group 4 demonstrated elevated expression of *phnB, phoD,* and *trpD*, suggesting active phosphorus cycling and auxin biosynthesis in this group. Group 7 showed enrichment of *cysN, cysD*, and *nifH*, indicating their roles in sulfur assimilation and nitrogen fixation. Groups 1 and 2, which were proficient in oil degradation, exhibited lower expression of plant growth-promoting bacteria (PGPB) genes, suggesting a trade-off between hydrocarbon degradation and plant-beneficial interactions. Groups 8 and 9 displayed moderate expression of *nifK, glna, phoH*, and *cysE*, indicating generalist communities with overlapping roles in PGPB and oil degradation. The heatmap reveals the specialization of microbial communities across environmental niches. Variations in hydrocarbon degradation among groups (e.g., Groups 1, 2, and 7) and (e.g., Groups 3, 4, and 7) demonstrate functional adaptations influenced by environmental conditions. These findings are crucial for managing microbial resources and restoring ecosystems in oil-contaminated mangroves.

### 3.7 Functional annotation based on KEGG orthologs (KOs) across groups

Comparative functional profiling across the nine study groups revealed distinct patterns in KEGG ortholog abundance, indicating environmental adaptation and microbial specialization (Table 5). The peptide/nickel transport system ATP-binding proteins K02031 (*ddpD*) and K02032 (*ddpF*) were the most prevalent, with Groups 9 (0.0073) and 7 (0.0077) exhibiting the highest levels, suggesting an increased demand for peptides and metal ions. The gene K00059 (*fabG*, OAR1), associated with fatty acid biosynthesis, demonstrated elevated expression in Groups 6–8, implying membrane biosynthesis in response to stress conditions. These observations suggest active lipid metabolism and membrane maintenance, with genes related to signal transduction. The methyl-accepting chemotaxis protein gene (K03406: *mcp*) exhibited the highest abundance in Groups 5 and 9, indicating enhanced microbial motility and nutrient sensing. Iron acquisition-related KOs, K02013 (ABC.FEV.A) and K02010 (*afuC/fbpC*), remained stable but elevated in Groups 6 and 7, suggesting iron-limited conditions.

**Table 5:** PERMANOVA results comparing microbial community composition across groups based on weighted and unweighted UniFrac distance matrices.

Comparison	R^2^	*F* value	*p* value
Comparison 1	0.614	12.73	0.012
Comparison 2	0.200	2.00	0.074
Comparison 3	0.776	27.66	0.007
Comparison 4	0.129	1.19	0.240
Comparison 5	0.208	2.36	0.025
Comparison 6	0.192	1.66	0.068
Comparison 7	0.429	4.52	0.001
Comparison 8	0.446	5.23	0.001
Comparison 9	0.410	3.82	0.001

The table presents R^2^ values (explained variance), F-values (test statistics), and associated *p*-values for each pairwise or grouped comparison. Higher R^2^ and F-values indicate greater separation between groups in terms of beta diversity. Statistically significant comparisons (*p* < 0.05) indicated meaningful differences in microbial community structure among the groups.

## 4 Discussion

Microbial communities in mangrove ecosystems play a crucial role in nutrient cycling and degradation of environmental pollutants, particularly hydrocarbons (Siddique et al., 2024). This study highlights how root proximity and geographic location influence the microbial diversity and functional potential in mangrove sediments subjected to petroleum hydrocarbon contamination.

Low-molecular-weight PAHs (LMW PAHs), including naphthalene and phenanthrene, are the most abundant PAHs in petroleum contaminated coastal sediments, due to their solubility and bioavailability (Alshemmari, 2021). This study found the highest concentrations of PAHs in sediments deposited near the mangrove roots compared to the bulk sediments 5 m away from the three study sites. The high concentrations of PAHs in the sediments near the mangrove roots at the three sites have implications for the microbial communities in these sediments, as there are specialized microbial populations that can degrade PAHs in the rhizosphere (Alsharif et al., 2024). Comparison of PAH levels between the studied locations showed that Sulaibikhat had the highest levels of total PAH followed by Shuwaikh and Al Khiran. High levels of PAHs found in the sediments of Sulaibikhat could be due to the location of the site close to several industrial and urban discharge areas. The site is situated near Sulaibikhat Bay, which receives water from runoff of the Kuwait City metropolitan area (Al-Ghadban and El-Sammak, 2005). The Al Khiran area, located in the southern part of Kuwait and far from most industrial activities, had lower PAH levels than the other two sites. The data from this study indicate that local human activities affect the chemical composition of mangrove sediments and thus may influence their potential use.

The predominance of naphthalene and phenanthrene in the sediment samples is particularly relevant for bioremediation, as these compounds are known to be readily degraded by a wide range of bacterial genera, including *Pseudomonas*, *Burkholderia*, and *Rhodococcus* (Tiralerdpanich et al., 2018). The presence of bioavailable PAHs in sediments suggests that indigenous microbial communities may have developed adaptive mechanisms for their degradation. This hypothesis was further explored in the subsequent sections through the isolation of PAH-degrading bacteria and the analysis of functional gene profiles. Overall, the chemical characterization confirmed that the mangrove sediments in Kuwait harbor significant PAH contamination, with spatial heterogeneity driven by both proximity to mangrove roots and geographic location, thereby providing a suitable context for investigating the bioremediation potential of resident microbial communities.

It is interesting to note that *Rhodococcus ruber*, the species isolated in this study, are known to degrade organic compounds such as high-molecular-weight PAHs (Krivoruchko et al., 2019). The isolation of *Acinetobacter soli*, which possesses alkane hydroxylase genes have also been reported to degrade n-alkanes, which are major fractions of crude oil (Li et al., 2025). The occurrence of these species in the same sediment samples as those from which the bacterial strains were isolated in this study suggests that there is a functionally redundant microbial consortium that can degrade a wide variety of hydrocarbons, thereby increasing the bioremediation potential of mangrove sediments in Kuwait.

The presence of oil modifies sediment microbial communities by exerting increased selection pressure on oil-degrading bacteria (Yu et al., 2022). In enrichment cultures, hydrocarbon-degrading taxa are favored, resulting in a reduction in alpha diversity in petroleum-contaminated soils, as evidenced by a decrease in the Shannon index from approximately 7.9 to 2.8 (Alghamdi et al., 2024). Mangrove rhizospheres sustain more diverse microbial populations than sediments, underscoring the role of root systems in facilitating coastal bioremediation processes. This study observed that microbial communities in mangrove rhizosphere sediments exhibit higher diversity and distinct taxonomic compositions than those in bulk sediments across three mangrove sites in Kuwait.

However, sediment samples from 5 m away from the mangrove plants, that is, the bulk sediments, exhibited lower alpha diversity. This may be due to the relatively homogeneous environment and possibly stressful conditions for the microbe’s growth (Das et al., 2024). This was also supported by the decline in the microbial richness with increasing distance from mangrove roots (1–5 m), as indicated by reduced OTUs and diversity indices, reflecting the diminishing influence of root-derived substrates and chemical gradients that support distinct consortia in proximity to roots (Bhattacharyya et al., 2015). In general, the diversity in the rhizosphere was higher than the diversity in the adjacent sediments, which is consistent with the rhizosphere effect (Haldar and Nazareth, 2018). The highest diversity values for the different locations were measured for the samples from Shuwaikh, whereas the samples from Sulaibikhat and Al Khiran exhibited lower diversity values, especially for the rhizosphere and nearby sediment samples. This was also reflected in the weighted UniFrac PCoA analysis, where the rhizosphere samples were clearly separated from the bulk sediment samples along PCoA1. This strong influence of the mangrove roots on the sediment microbial communities is well established and the rhizosphere of mangroves can be considered a “hotspot” of high microbial activity and diversity (Alsharif et al., 2024). The diversity in the sediments from Shuwaikh, which are situated in the vicinity of an urban area, was higher than expected, possibly due to the moderate level of disturbance, which can create new niches for additional taxa (Li et al., 2019). Site-specific pairwise comparisons were consistently grouped by location (PERMANOVA, *p* < 0.05), suggesting the influence of environmental and anthropogenic factors (Wang and Shao, 2013). Cotta et al. (2019) has reported archaeal richness in the sediments of the Sundarbans at Godkhali, Bonnie Camp, and Dhulibhashani.

The enrichment of *Vibrio* and *Photobacterium* in the rhizosphere samples of comparison 3 is related to the capability of these bacteria to degrade rhizosphere regions (Das et al., 2024). Previous studies have shown the presence of *Vibrio* species in the rhizosphere of plants growing in mangrove ecosystems (Tiralerdpanich et al., 2018). Studies have also established its role in degrading hydrocarbons. The preponderance of *Anaerolineae* in bulk sediment samples of Group 3 of comparison 2 is attributed to their characteristic fermentative mode of growth with ability to degrade anoxic environments characteristic of deeper sediment layers (Haldar and Nazareth, 2018). Interestingly, cyanobacteria found in rhizosphere samples suggest the support of the growth of heterotrophic bacteria by fixing nitrogen and providing primary end products (Haldar and Nazareth, 2018).

Additionally, interesting organisms have been detected as *Sulfurovum* in the bulk sediments of group 7 (comparison 7) which indicates sulfur cycling in the above anoxic zone (Alsharif et al., 2024). *Sulfurovum* species are sulfate reducers that use organic matter for their energy requirements and are thus typical of the above-mentioned zones in mangrove sediments. In another study, the presence of *the Thalassospira* genus, which has the potential to degrade aromatic hydrocarbons, was reported. These specialized organisms have been detected in the bulk sediments of Group 9 (comparison 6) (Tiralerdpanich et al., 2018).

The rhizosphere exhibited an enrichment of marine copiotrophs (*Marinomonas, Vibrio*, *and Photobacterium*) and taxa associated with nutrient cycling (*Rhodobacteraceae and Halomonas*), which are recognized for their metabolic adaptability in microtoxic environments (Bhattacharyya et al., 2015). In contrast, non-rhizospheric sediments displayed higher abundances of unclassified bacteria, *Anaerolineae*, *Sulfurovum*, and *Robiginitalea*, suggesting their role in organic matter degradation in reduced root zones. The presence of hydrocarbon-degrading taxa (*Caproiciproducens*, *Desulfotignum*, *and Sulfurimonas*) and sulfur-metabolizing organisms indicates microbiome adaptation to local conditions (Thatoi et al., 2012; Chen et al., 2019). Proximity to roots enhances diversity and supports functional microbial communities that facilitate nutrient transformation and pollutant resistance in mangrove habitats. A wide variety of species have been identified in the mangroves, which includes *P. alcaligenes, P. mendocina, A. borkumensis, D. vulgaris, D. desulfuricans, E. coli, P. denitrificans*, and *S. putrefaciens*, in mangrove soils, with *P. alcaligenes* demonstrating the ability to degrade PAHs (Chen et al., 2019). Microbial hotspots have been observed in the rhizosphere and rhizoplane, indicating an increase in bacterial diversity (Zhuang et al., 2020). Investigating root-associated microbiomes is crucial for understanding the resilience and functionality of mangrove ecosystems, emphasizing the intricate interactions within microbial communities (Alongi, 2014).

Hydrocarbon degradation and PGPB genes co-occur in several groups of organisms. In Shuwaikh rhizosphere sample (Group 1), the ability of microbes to degrade oil and promote plant growth simultaneously could be of great interest for the bioaugmentation of oil-polluted mangrove ecosystems. These bacteria are able to promote plant growth by fixing nutrients and protecting roots from toxic effects of pollutants; in return plants provide these bacteria with stable carbon source and suitable habitat (Alsharif et al., 2024). Genes for the production of PGPB, such as siderophores (*gltB*, a glutamate synthase involved in siderophore biosynthesis), were found to be expressed at higher levels in Groups 1, 4, and 7. Siderophores are iron-chelating compounds, produced by bacteria to acquire iron (Gupta and Gopal, 2008). This micronutrient is essential for most bacteria; however, in most aerobic environments, ferric iron is present in an insoluble form and is therefore not available to bacteria. Enrichment of genes for siderophore production in rhizosphere bacteria can indicate their role in improving iron availability for plants, thus promoting the growth of mangroves under stress conditions. Some of the genera identified in the study are known to degrade PAHs and include sulfur metabolism genes and, together these findings suggest a role for the root environments of these mangroves in the microbial bioremediation of oil in contaminated coastal systems and the use of the communities in environmental monitoring (Zhuang et al., 2020).

Functional annotation has identified key genes involved in hydrocarbon degradation in various bacterial groups. Groups 3, 5, and 9 exhibited elevated *alkB* expression, which is crucial for the degradation of aliphatic hydrocarbons (Haritash and Kaushik, 2009; Fenibo et al., 2023). In contrast, groups 2 and 4 were enriched with *nahA*, *nahB*, and *phnABC*, which are essential for PAH catabolism. Groups 1 and 6 contained genes associated with toluene degradation, namely *todC1* and *bedC1*, respectively. The high abundance of *nif* genes in the rhizosphere is consistent with the known association between nitrogen-fixing bacteria and plant roots, where root exudates provide a carbon source for the diazotrophs in exchange for fixed nitrogen (Das et al., 2024). Phosphate solubilization is a critical PGPB trait, as phosphorus is often present in mangrove sediments in insoluble forms that are unavailable to plants (Haldar and Nazareth, 2018).

KEGG ortholog analysis has revealed metabolic specialization in mangrove sediment (Das et al., 2024). Groups 7 and 9 demonstrated a high abundance of ABC transporters (K02031, K02032) for peptide and metal ion translocation (Wilkens, 2015; Rémy et al., 2018). Proteins K02003 and K01990 are present in mangroves with varying organic loads and salinity (Ribeiro et al., 2018). The gene K00059 (*fabG*) indicates lipid metabolism stress responses (Capra and Laub, 2012), which was prevalent in groups 6–8. K02483 (*OmpR*) and K02488 (*pleD*) coordinate the responses to osmotic changes (Römling et al., 2013). K21023 (*mucR*) and K13590 (*dgcB*) enhance biofilm formation, with Group 3 showing advantages in high-shear stress areas (Porter et al., 2011). Chemotaxis genes K03406 and K13924 in Groups 5, 9, and 3 suggest nutrient gradient habitats that facilitate microbial colonization (Andrews et al., 2003). The iron-acquisition genes K02013 and K02010 were present across groups, with higher levels in Groups 6–7, indicating high-affinity absorption needs. Microbes require iron regulation because of its limited bioavailability in anoxic sediments (Frost et al., 2005). Genomic plasticity enables microbial adaptation to contaminated environments, as evidenced by the increased transposase gene K07497 in Groups 6–8. Heavy metal tolerance is common in mangroves, with genetic mobility facilitating the dissemination of this trait (Toussaint and Chandler, 2011; Bag et al., 2022). The KO-based profile revealed functional variations among mangrove zones and core microbial capabilities, reflecting tidal patterns, organic matter, redox potential and anthropogenic activities.

However, our analysis has some limitations. First, the functional genes in the sediment microbes were predicted using PICRUSt from the 16S rRNA gene sequences. Since the method projects metagenomic content from 16S rRNA genes to whole genomes of reference microbes, it fails to capture the complete gene repertoire of unknown or novel microbes, especially those with distant relative genomes that have not been fully sequenced and analyzed yet (Langille et al., 2013). In our taxonomic analysis, a considerable proportion of bacteria could not be assigned to known species or classes, and their functional potential remains to be discovered. This is particularly true for the culturable PAH-degrading bacteria isolated and analyzed in this study. To enrich PAH-degrading bacteria, several key growth conditions were provided, and Pseudomonas was favored as a model group of fast-growing, highly versatile, copiotrophic bacteria that can degrade hydrocarbons. Thus, the study mainly contains slow-growing and highly specialized obligately or facultatively anaerobic PAH-degrading microbes, which are difficult to culture and isolate in the laboratory. In addition, there were only two sampling times in this study, conducted in September 2023 and March 2024. Because there are many environmental factors, such as temperature, salinity, and tidal actions, that change frequently over seasons, the two times might not be sufficient to illustrate the complete seasonal variations of the microbial community and its functional potential in the studied sediment environment.

However, several aspects of this study require further investigation. First, the expression of the predicted genes *in situ* by the corresponding organisms must be proven by metatranscriptomics and/or metaproteomics. Furthermore, gene expression needs to be seasonally monitored to determine the possible environmental factors affecting the pathways for the hydrocarbons to degrade (Tiralerdpanich et al., 2018). Experiments to check the interactions of single bacterial species that have been isolated as potential PAH degraders and their degradation capability in consortia could clarify their additional value for overall PAH degradation. Degradation studies using microcosms filled with sediments from all three investigated sites and testing inoculated consortia and different combinations of the individual bacteria could help specify the best consortia as well as an optimal design of an application for field experiments.

## 5 Conclusion

This study reports a comprehensive metagenomic investigation of mangrove sediment microbiota from three sites across Kuwait, highlighting its fundamental roles in the degradation of Polycyclic Aromatic Hydrocarbons (PAHs) and promotion of plant growth. The sediments retrieved from the immediate vicinity of the mangrove roots harbored microbiota distinct from those encountered within the rhizosphere-free sediment environments. Moreover, the spatial geography of microbial communities in sediments sampled across different sites was an important factor influencing microbial profiles. Furthermore, we noticed that the microbiota degrading PAHs from these sites possess unique functional genes, such as those involved in the degradation of both alkane- and aromatic-hydrocarbons, with the alkane-degrading *alkB* gene enriched within the Sulaibikhat and Al Khiran groups. In contrast, genes for the degradation of PAHs, including the well-studied *nahA* and *phnA*, were predominant in very high abundance within the Shuwaikh rhizosphere sediment community. Strikingly, several important genes involved in promoting plant growth, including those necessary for both nitrogen fixation and phosphate solubilization, were found to co-occur in the same major microbial groups that also degrade various types of petroleum hydrocarbons. This highlights that these major hydrocarbon-degrading sediment microbial groups play crucial roles in supporting the growth and development of mangroves and detoxifying toxic petroleum hydrocarbons. Thirty bacterial strains were isolated as potential candidates for future implementation of environmental remediation, including members of *Pseudomonas* sp., *Pseudomonas stutzeri*, and well-known PAH degraders, including *Rhodococcus ruber*.

Future studies should investigate the expression of functional genes in sediment microbial communities through metatranscriptomics and test the potential of isolated bacteria to degrade petroleum hydrocarbons in synergistic microcosm experiments. Genome-resolved metagenomics should also be performed to unlock the unexplored metabolic potential of the large proportion of unclassified bacteria in these sediments to harness their bioremediation potential for the restoration of oil-contaminated mangrove habitats in Kuwait and the wider Arabian Gulf region.

## Data Availability

The datasets presented in this study can be found in online repositories. The names of the repository/repositories and accession number(s) can be found at: https://www.ncbi.nlm.nih.gov/, PRJNA1434151.
